# Transfer Learning-Based Approach for Thickness Estimation on Optical Coherence Tomography of Varicose Veins

**DOI:** 10.3390/mi15070902

**Published:** 2024-07-10

**Authors:** Maryam Viqar, Violeta Madjarova, Elena Stoykova, Dimitar Nikolov, Ekram Khan, Keehoon Hong

**Affiliations:** 1 Institute of Optical Materials and Technologies, Bulgarian Academy of Sciences, 1113 Sofia, Bulgaria; vmadjarova@iomt.bas.bg; 2 Faculty of Information Technology and Communication Sciences, Tampere University, 33720 Tampere, Finland; 3 Department of Vascular Surgery, Sofiamed University Hospital, 1797 Sofia, Bulgaria; dimn@mail.bg; 4 Department of Electronics Engineering, Aligarh Muslim University, Aligarh 202001, India; ekhan67@gmail.com; 5 Electronics and Telecommunications Research Institute, 218 Gajeong-ro, Yuseong-gu, Daejeon 34129, Republic of Korea; khong@etri.re.kr

**Keywords:** varicose vein, optical coherence tomography, segmentation, thickness

## Abstract

In-depth mechanical characterization of veins is required for promising innovations of venous substitutes and for better understanding of venous diseases. Two important physical parameters of veins are shape and thickness, which are quite challenging in soft tissues. Here, we propose the method TREE (TransfeR learning-based approach for thicknEss Estimation) to predict both the segmentation map and thickness value of the veins. This model incorporates one encoder and two decoders which are trained in a special manner to facilitate transfer learning. First, an encoder–decoder pair is trained to predict segmentation maps, then this pre-trained encoder with frozen weights is paired with a second decoder that is specifically trained to predict thickness maps. This leverages the global information gained from the segmentation model to facilitate the precise learning of the thickness model. Additionally, to improve the performance we introduce a sensitive pattern detector (SPD) module which further guides the network by extracting semantic details. The swept-source optical coherence tomography (SS-OCT) is the imaging modality for saphenous varicose vein extracted from the diseased patients. To demonstrate the performance of the model, we calculated the segmentation accuracy—0.993, mean square error in thickness (pixels) estimation—2.409 and both these metrics stand out when compared with the state-of-art methods.

## 1. Introduction

The human vascular system is important as it nourishes the tissues and organs by transporting the blood containing essential nutrients and oxygen. Three main types of vessels are the arteries, veins, and capillaries. The unique physiology of the vascular walls imparts them with essential mechanical characteristics required for proper circulation of the blood. However, any changes incurred in the structure, composition, or surroundings of the veins can cause malfunctioning in the vascular system. Alterations in vascular structures can result in diseases such as chronic venous disease (CVD) or varicose veins [1]. The remodeling of venous walls leads to alterations in the lumen diameter and wall thickness, along with other changes in mechanical properties [2,3,4]. In such cases, thorough understanding of the venous properties is desired to treat the veins adequately. Many studies analyze the vein diameter and wall thickness to comprehend venous diseases and the associated remodeling [2,5,6,7,8,9]. In most of these studies, the diameter and the thickness are cardinal elements required for modelling the physical properties in veins.

Tissue engineering and science of the biomaterials have been focusing on the study of vascular substitutes to recapitulate their physical properties. Possible solutions include replacement using synthetic prosthesis (SP), autografts (AGs) or tissue engineered vessels (TEVs). These substitutes should encapsulate certain mechanical characteristics to replicate the natural functioning of the vessels. Even with tremendous research and development in this field, AGs using the saphenous veins stand as the most compliant solution.

Several tests are performed to study, design and analyze these substitutes like tensile stress–strain, compression, burst pressure, stress–relaxation, creep-testing, to name a few. Geometrical estimates of shape along with diameter/thickness measurements are crucial for such testing methods or experiments. Practically in laboratory environments, such measurements are done using a vernier caliper whose accuracy is of the order of a few millimeters. But in certain studies, such a range of accuracy and precision is not enough for investigation and modeling purposes. To study the effects of certain diseases, the variations may be in the range of a few micrometers. Seemingly different, but demandingly the same, all the analysis and testing techniques require methods to study the shape and physical characteristics of the vein [10]. Hence, one of the enabling steps of the physical characterization problem in veins is the shape analysis along with a highly accurate thickness measurement. Furthermore, an important aspect is the estimation of the precise location of the site while performing comparative or progressive experimental studies. The shape in form of segmentation maps aids the process of fixing the sites of measurement. This exemplifies the need for a parallel approach to obtain a segmentation map along with thickness values.

The non-uniform variations in the wall thickness require establishing a 3D model with thickness maps to perform an analysis. With the 3D OCT imaging technology, in our research SS-OCT, it is possible to acquire volume data on human veins in a contactless manner with high precision. It can be used to visualize and model the physical properties of the vein. A growth has been seen in using OCT for several pre-clinical applications to analyze the structural details in different kinds of medical samples [11]. Once the dataset is acquired, doctors can manually delineate the venous surfaces and measure several physical parameters. However, the manual segmentation for such acquired volumes requires expertise, and it is a time-consuming and exhaustive process. Moreover, such segmentation is not possible in real-time applications. Hence, it is crucial to establish artificially intelligent models that can infer the shape and size accurately and precisely for the veins, especially the thickness, with less human intervention.

In this work, we advance an automated model capable of segmentation and thick-ness estimation with a view to better understand the properties of veins by analyzing their shape and estimating their thickness at the same time. An ensemble model—TRansfer learning based approach for ThicknEss Estimation (TREE) —is proposed in this work. It is a dual prediction model that predicts the segmentation map along with the thickness values for OCT images of varicose veins. It has an encoder–decoder type of architecture that stands on the cardinal stem of an encoder followed by bifurcation into two branches containing decoders. The two decoders learn, to generate a segmentation map and the thickness value of the veins, respectively, using the output of the encoder which is trained only once to learn the semantic features of the veins. Explicitly, the model is first trained to learn global and local cues essential for segmentation using an encoder and segmentation decoder. Next, the encoder layers are frozen with the weights learnt during the previous step, and the layers of the second decoder are trained to predict the thickness values. At the divergent node, the encoder output is rich with global semantic details. This block called a sensitive pattern detector (SPD) incorporates atrous and depthwise convolution layers arranged in a special manner described in Section 3. The former convolution allows the model to capture long-range dependencies while the latter makes the network more efficient. The novelty in the work lies in proposing a dual prediction model which gives both shape and thickness information. It is based on self-transfer learning that allows a flow of enhanced features from the segmentation part of the model into the thickness prediction part where the features are further refined to converge the model.

The remainder of this paper is organized as follows: Section 2 focuses on the background; Section 3 describes the materials used and the method proposed; Section 4 presents the results of the proposed work and performs a comparison; Section 5 provides discussions and the conclusions regarding the proposed work.

## 2. Background

Many advancements have been made in the field of biomedical image processing with the automated image segmentation and interpretation methods for improved diagnosis and studies. Among the classical methods, graphs-based [12,13,14], texture details [15], and mathematical morphology [16,17] are commonly used. However, they are usually fraught with errors and inaccuracies due to human errors and the limitations of the human visual system. Indeed, the contributions by deep-learning [18] based artificially intelligent architectures have established them as highly accurate and powerful segmentation models [19,20,21,22,23,24,25,26,27,28,29]. Several layers of transformations were employed in the deep-learning models to obtain optimal features during the training process to achieve fine-tuned results. Many novel deep-learning architectures followed by their variants like UNET [19,20,21], residual-connection based models [22,23,24,25], PSPNET [26], SEGNET [27], and DeepLab [28,29] are known for their outstanding performances in the field of image segmentation. In particular, UNET [19] has been widely used in medical image segmentation due to its capability to leverage even with a limited number of image samples. It is an encoder–decoder type architecture where information exchange occurs through skip connections. Nevertheless, the model needs to tune large number of parameters making it computationally complex. Residual connections [22], well known for their effectiveness against vanishing and exploding gradients in deeper networks, were incorporated into UNET [19] architecture to make it more robust in the RESUNET [23]. SEGNET [27] generates sparse feature maps, wherein it uniquely upsamples on the decoder side by using the indices from the max-pooling layers on the encoder side. It has the drawback of mis-classification as there is no exchange of semantic cues from the encoder towards the decoder side, which are important as carriers of global features.

DeepLab3+ [29] uses encoder–decoder architecture for feature extraction at multiple scales, depthwise separable convolution for more efficiency and parallel atrous convolution in addition to a spatial pyramid pooling module for sharper boundaries and better field-of-view. Furthermore, PSPNET [26] also uses pyramid pooling to extract multi-scale information. Both these methods keenly focus on extracting global contextual information using different grid scales but at the same time neglect the importance of low-level spatial cues. These models, described above, mainly emphasize features based on 2-D local spatial characteristics and lack depth-resolved features. They are unable to make complete use of spatial and depth information especially in three-dimensional data. Hence, there remains room for improvement of accuracy in these segmentation methods.

A commonly used approach to estimate the thickness in various biomedical images is to process the segmentation maps by employing a mathematical formulation for thickness calculations [30,31,32]. A segmentation-free approach was proposed by Mariottoni et al. [33] to estimate the thickness value of the retinal layer in OCT scans. Though the approach is unique for thickness calculation, the absence of a segmentation map does not allow a performing shape analysis to interpret the properties of veins.

The proposed model TREE has a modified encoder–decoder style of architecture based on UNET [19]. The dual prediction model allows us to predict both the shape and thickness information with greater accuracy. Moreover, the thickness is independent of the segmentation map (generated from the segmentation model), which can lead to error accumulation. It is based on self-transfer learning where it learns features during one part of model optimization (segmentation) and fine-tunes them further to converge the thickness prediction part of the architecture. The model accomplishes both the predictions with greater accuracy and efficiency. The main contributions and highlights of this work are as follows:

1. Problem Formulation: The gap in studies, related to veins, demands models that can predict physical features like the shape, inner–outer surfaces, thickness, etc. Hence, we formulate the objective as a model design problem where we optimize the model parameters to predict the shape and the thickness of veins using OCT images.
2. Methodology: We leverage an ensemble model that allows the encoder–decoder style architecture to learn the segmentation and thickness features in veins sequentially through transfer learning using residual connections and atrous and depthwise convolution in a special manner to improve accuracy.
3. Dataset: We use a dataset of saphenous varicose veins based on SS-OCT to evaluate its physical features.
4. Evaluations: We assess the model by performing experiments and evaluating several metrics suitable to judge the relevant physical features extracted. The model is compared with other state-of-art methods for fair evaluation.

## 3. Materials and Method

This section provides a detailed description of the proposed architecture TREE. Firstly, we describe the image acquisition system SS-OCT, followed by a description of the proposed model and its components.

The dataset used was acquired by SS-OCT by Optores GmbH device, which belongs to the Fourier domain (FD) category of OCT systems. The light source was a Fourier domain mode locked (FDML) swept laser [34,35], at a sweeping frequency of 1.6 MHz per A-line. The central wavelength of the FDML was 1309 nm, with a bandwidth of 100 nm. The OCT system was a fiber-based interferometer where the interference signal corresponding to each sweep of the laser is recorded by a photo detector over time. The 3D scanning is carried out by a Galvano scanner-mirror that scans in X and Y directions. Each recorded scan of the swept source is Fourier transformed to generate the A-scan—the depth profile (Z direction) at a single point. B-scan images were composed of A-scans at different positions in the X-direction, thus representing the X–Z plane. The 2-D representation of the veins, namely B-scans, is used in this work after denoising [36] them. They serve as input to the prediction model TREE described below. No additional enhancement techniques were applied to the dataset. Further details about the complete dataset, image resolution, data partitioning, augmentation, and training can be found in Section 4.

**Figure 1:**
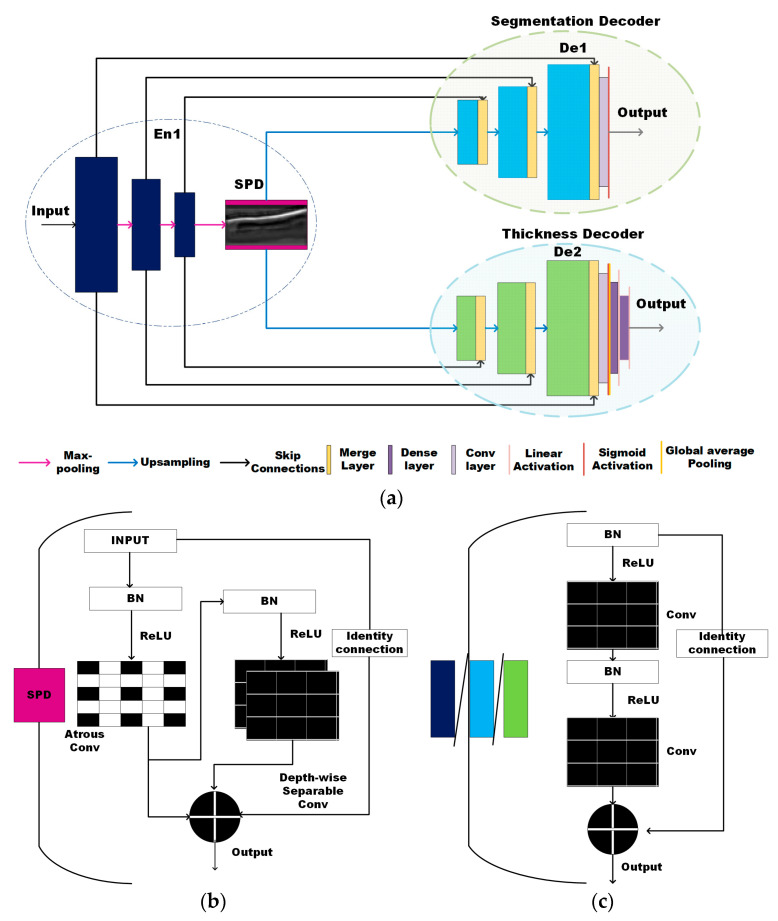
(**a**) Block diagram of the proposed model showing overall architecture of the encoder and two decoders (**b**) Sensitive pattern detector module (**c**) Encoder or decoder blocks with layers and flow of information.

To facilitate the use of OCT for automated segmentation and thickness calculation of the veins, this work introduces the architecture named TREE. It deploys the B-scans to perform this prediction of the venous images. Figure 1a illustrates the proposed model having the single-encoder dual-decoder style architecture inspired by the UNET [19]. The high-level feature map at the bottleneck between the encoder and the decoder of UNET [19] model plays an important role in prediction of the outputs. TREE makes unique use of these bottleneck features for the transfer of information from a segmentation to a thickness estimation model. It has three main components: one encoder and two decoders. The first decoder (De1) generates the segmentation map by classifying the pixels, while the second decoder (De2) measures the thickness of the vein. The encoder (En) is connected to both decoders, forming two branches, one with En-De1 and other with En-De2. The output of the encoder is forwarded to a bottleneck module (depicted in pink in Figure 1a) that is used by both decoders. This bottleneck module leverages the model, by giving more attention to the region of interest in each B-scan.

The encoder (En) and decoders (De1, De2) contain three main blocks marked in dark blue and light green (as rectangular boxes), respectively, in Figure 1a. The block (En/De) is illustrated in Figure 1b showing the stacked layers. Each block performs two convolution operations along with batch normalization (BN) and activation using rectified linear units (ReLU). A residual connection [23] is used to attain consistent training with the increasing number of layers by adding the input to the last layer of the block (output). These identity connections help to address the problem of gradients effectively. Unlike the standard UNET [19] model, the TREE incorporates batch normalization in all the blocks [37] along with the introduction to a sensitive pattern detector (SPD) module between the encoder and decoder as shown in Figure 1a,c. All the convolutions used in encoder/decoder blocks along with the SPD utilize a 2D-kernel of size 3 × 3, stride 1 × 1; padding is set to the same. The SPD is placed at the bottleneck of the encoder–decoder branches. At the confluence, the two decoder branches arise. After every encoder block, we place the max-pooling layer to reduce the feature maps. The final encoded feature map is then fed to the SPD module elaborated in Figure 1c to extract the features necessary for predicting thickness and segmentation maps. Then, the output of this module enriched with global information is upsampled and passed through the decoder blocks. The max-pooling and upsampling operations extract features at varying resolutions. The global information is fused with localization information using the intermediate connections that allow the flow of information at the same scales. Let the input be Χ∈ R^(H × W × D), where H, W, and D represent the dimension of the feature map.

The regular convolution can be written as:

(1) $${RegularConv:\;\sigma (X,W_{g})}_{\left(i,j\right)}=\sum_{k,l,m}^{K,L,M}X(k,l,m)*W_{g}(i+k,j+l,m),$$

where (*i, j*) is the position on input image or feature map (input), $W_{g}$ is the filter with dimensions (*k, l*) and *m* is the number of channels in input. Following from the regular convolution, the overall process of each encoder (or decoder) block can be expressed as:

(2) $$Y_{en}=\sigma (ReLU(BN(\sigma (ReLU(BN(X)),W_{g}),W_{g})+\sigma (ReLU(BN(X)),W_{g}),$$

The output from each encoder block is downsampled using the max-pooling technique to obtain global features defining the boundary details. This model uses the bottleneck module comprising the atrous convolution and depthwise separable convolution for enhanced semantic feature extraction inspired by our previous work [38]. The regular convolution completes the feature learning process with an information exchange bounded to a small patch depending on the size of the kernel (usually 3*3). This creates a problem when segmentation masks have long-range spatial relations. The morphology of the veins manifests itself in elongated structures unlike other bio-medical segmentation targets like cancer or tumor cells. To capture such dependencies, feature maps having several layers in depth are acquired using the depthwise separable convolution. As seen in Figure 1c, the module contains a depthwise separable and atrous convolution in parallel along with BN and ReLU activations. The outputs of both the convolutions are added to the last layer along with the residual connection to obtain the final feature map. In contrast to Opto-UNet [38], we used dilation in the DS convolution layer to maintain opaqueness (where the weights of the filter lie) and transparency (where the holes exist) of the atrous layer with a depthwise layer as they are added. This unique stack of multi-purpose convolution layers improves the receptive field along with enhanced spatial and depth information without burdening the number of model parameters. They produce disentangled feature layers allowing the network to recognize the sensitive area of images, called the SPD. The atrous convolution allows an exchange of information over a larger area within an individual layer rather than stacking layers to capture spatial dependencies. The atrous convolution on the individual layer (2-D) of input Χ can be expressed as:

(3) $${Atrous:\;\alpha (X,W_{g})}_{\left(i,j\right)}=\sum_{k,l}^{K,L}X(i+rk,j+rl)*W_{g}(k,l),$$

where *r* is the dilation rate, (*i, j*) is the position on the input image or feature map (input), $W_{g}$ is the filter with dimensions (*k, l*). The atrous convolution injects holes between the weights of filters. This helps the model to learn features from a wider field of view while retaining the same number of model parameters as in a regular convolution.

The depthwise separable (DS) convolution incorporates a depthwise and pointwise convolution to learn better representation in depth and space simultaneously. First, each channel is convolved with a separate kernel to obtain the spatial information; then, at each spatial position the information is combined for depth channels using the pointwise convolution. The pointwise convolution weighs all the depth maps at a single spatial position. Effectively, it prevents mixing of the information from several depth channels along different spatial positions generating a deeper information-enriched network. It was demonstrated in [39,40] that DS convolution improves computational efficiency by using a reduced number of parameters without compromising accuracy. The two-step pointwise and depthwise convolution can be described mathematically as:

(4) $${Pointwise:\;P(X,W_{g})}_{\left(i,j\right)}=\sum_{m}^{M}X(m)*W_{g}(i,j,m)$$

(5) $${Depthwise:\;\partial \left(X,W_{g}\right)}_{\left(i,j\right)}=\sum_{k,l}^{K,L}X\left(k,l\right)*W_{g}\left(i+k,j+l\right),$$

The combined expression for depth-wise separable convolution is as follows:

(6) $$SepDepthwise:\;\delta {\left(X_{p},{X_{\left(d\right)},W}_{g}\right)}_{\left(i,j\right)}=P_{\left(i,j\right)}\left(\left(X_{p}\partial {\left(X,W_{g}\right)}_{\left(i,j\right)}{(X}_{\left(d\right)},W_{g}\right)\right),$$

The addition of the two types of feature fields determined uniquely in Equations (4) and (5) helps us to extract more versatile features contributing to both aims; venous layer segmentation along with thickness calculation. The overall operation of the block can be written as:

(7) $$Y_{bb}=\alpha (ReLU(BN(X)),W)+\delta (ReLU(BN(X)),W)+\varphi \left(BN\left(X\right),W\right),$$

where *ReLU* is the activation function, and *α*, *δ*, *φ* perform the atrous, depthwise separable (with dilation = 2), and regular convolution (identity mapping), respectively. This SPD module generates maps that aid the learning process for segmentation and thickness by exchanging global-level features. These feature maps are upsampled next, to obtain pixel-level precision for desired thickness estimation and segmentation. The De2 has similar encoder/decoder blocks marked in blue (illustrated in Figure 1b) with three dense layers activated linearly and stacked at the end of the decoder.

**Figure 2:**
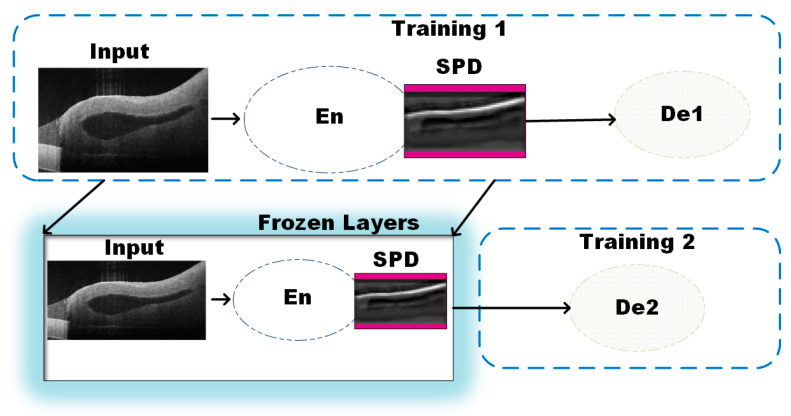
Block diagram representation of the training for the proposed model using the Encoder (En) and the two decoders (De1 and De2).

The learning of salient features is conducted in a sequential manner as shown in Figure 2. During the training stage, first the branch 1 (En-De1) is trained to obtain the segmentation map, followed by training of the second branch (En-De2) to obtain the thickness value of the vein (B-scan). The training for En-De1 allows precise pixel level segmentation. Further, a feature map from the encoder followed by the SPD module is extracted and fed as an input sequence to the second decoder (De2) to effectively train the second branch (En-De2) for thickness estimation. During the second training phase, the weights for the En and bottleneck module are obtained from the previous segmentation learning and only decoder-De2 is made to learn new features for precise thickness estimation. To adapt the model for the regression task, the second decoder (De2) has three dense layers with linear activations, different from the De1 whose last layer is a convolution with a sigmoid activation. The unique approach TREE allows the decoder to learn more localized features required to estimate the thickness of veins and compensates for the segmentation inaccuracies in estimating the thickness.

## 4. Experiments and Results

This section describes the specifications of the varicose vein dataset, implementation details, performance metrics, and comparative evaluations with the state of the art. The proposed model is compared with state-of-art models, namely UNET [19], PSPNET [26], RESUNET [23], DeepLab3+ [29], and SEGNET [27], using different performance indicators.

The dataset used [41] in this study comprises B-scans obtained using SS-OCT by Optores GmbH. The samples were taken from the Hospital Sofiamed, Sofia, Bulgaria, with the informed consent of the patients suffering from varicose vein disease. An approval by the Ethical Committee of the Bulgarian Academy of Sciences (permission 1-44/11.06.2021) was granted. The study was performed in accordance with the tenets of the Declaration of Helsinki of 1975, revised in 2013. Informed consent was obtained from the patients before the surgical procedure. The dataset contained five volumes having 1300 B-scans, collected from four patients. Each B-scan had 1024 A-scans and was cropped and resized to a resolution of 512 × 256 due to computational limitations and the region of interest. All the images were denoised using the method proposed in [36]. They were normalized in the range [0, 1]. The experiment was performed on the system with AMD Ryzen 7 as the PC processor having an NVIDIA GeForce RTX 3060 graphics card, 32 GB RAM and 1TB SSD from Dell Inc., Round Rock, TX, USA with Keras API integrated in a TensorFlow 2.0 environment. The optimizer used was Adam with a learning rate of 0.0001, a batch size of 16, and a maximum of 200 epochs. These training parameters were kept the same for all the other state-of-the-art models to perform a fair comparison.

**Figure 3:**
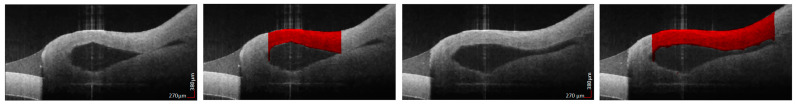
Original images and their segmentation map overlaid on original image.

The data from four volumes were organized randomly into training, validation, and testing groups having a ratio of 70%, 20%, and 10%, respectively. The dataset included different veins with varying shapes and sizes represented by each B-scan. To enforce diversity, the scans were geometrically transformed using translation and flipping during learning to augment the dataset. Translation was 20% for both height and width of image. Flipping was conducted for horizontal and vertical modes. The ground truth as segmentation maps was delineated manually under a medical expert (D.N.) in the field of vascular surgery, as shown in Figure 3. Some regions of the veins were excluded during annotation for generating segmentation masks due to ambiguity in distinguishing the border pixels. It is to be noted that only the top 2D-layer of the vein is used, as the lowest surface is not visible due to the limitations of depth penetration in the OCT. This was followed by a ground truth thickness calculation for each row axially, which we call a thickness map.

The proposed model was trained in a sequentially supervised fashion. Firstly, the encoder (En) and decoder (De1) ensemble were trained for performing the segmentation using binary cross-entropy (BCE) loss (Loss1). This was the base-learner which minimizes the loss between the manually annotated mask and predicted output where each pixel is mapped to 0 or 1 (foreground and background). To predict the mean thickness value of the vein, a mean square error (MSE) was used as a second loss function (Loss2) for En-De2. The predictions of the bridge block previously trained for segmentation that contained shape information were used as the input tensor to De2. Motivated by segmentation work [42], the BCE loss was used as the loss function to train the base–ensemble model.

**Figure 4:**
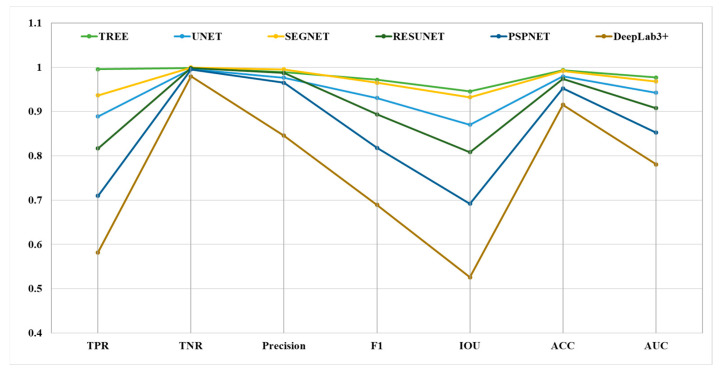
Segmentation metrics are represented as line plots for comparison of different models represented by each line with metric name and corresponding values on x-axis and y-axis, respectively.

The proposed model was evaluated to assess its performance and compare with well-known state-of-the-art methods mathematically using performance indicators. namely Accuracy, AUC, Intersection over Union, TNR, Recall, Precision, and the F1 score described in Table 1. This table includes two metric groups: spatial and probabilistic where the significance and formulas of the metrices are defined. The TNR indicates how well the model performs for a pixel outside the target group, whereas recall identifies the actual positives. Both are complementary to each other and helps to assess the performance of the model. IOU is a highly balanced and versatile metric as it incorporates both false positive and false negatives giving the overlap between predicted and ground truth images. Precision is crucial in applications where false positives are problematic, as it indicates that maximum number of pixels identified as target class are correct. To calculate F1 score, precision and recall are combined which gives a balanced measure that is highly significant when dealing with imbalanced datasets. ACC provides the over-all status of predictions but it might be mis-leading in case of data imbalance. AUC provides the model’s discriminatory property and allows for better comparative analysis. Hence, the combination of these metrics allows unbiased and precise estimate of a model’s segmentation performance. For comparison, we choose five state-of-art methods: UNET [19], SEGNET [27], RESUNET [23], PSPNET [26] and DeepLab3+ [29]. All the models, were trained in a similar manner like the proposed model using the same number of epochs, optimizer, learning rate, and batch size as mentioned previously. To analyze the experimental results of segmentation, these metrics were shown with the help of the line plots in Figure 4 for fair comparison. TREE performs better as compared to state-of-art methods for all the indicators except in precision metric for which only the SEGNET [27] model performs slightly better.

The thickness calculation was performed for ground truth and the state-of-art models as described below. To train TREE, the ground truth thickness of B-scans was estimated using the ground truth segmentation maps. And for predicting thickness using the state-of-the-art models UNET [19], SEGNET [27], RESUNET [23], PSPNET [26], and DeepLab3+ [29], we use their segmentation outputs. The pixels were segregated into a top layer and a bottom white layer in the segmentation map. Let the segmentation map be represented using the Matrix $M_{I\times J}$ where each element is represented as $m_{ij}$ where *i* and *j* represent the row and column, respectively. The layer 1 and 2 are the top and bottom layers of the vein, having a value 1 ($\hat{y}=1)$ for the topmost and bottom-most in respective columns belonging to the region of interest.

(8) $$C_{j}={\left[m_{1j},m_{1j},\ldots \ldots \ldots m_{I\times j}\right]}^{T}$$

(9) $$E^{k}=[e_{1},e_{2},\ldots \ldots \ldots e_{n}]$$

Let any arbitrary column of image matrix $C_{j}$ is given in Equation (8). and the edge of the region of interest of vein $E^{k}$ is a vector of size 1× n, expressed in Equation (9) and k ∈{Layer1, Layer 2}. The coordinates of the Layer 1 (L1) and Layer 2 (L2) in column $C_{j}$ can be calculated as:

(10) $$B_{j}=\arg\mathrm{min i}C_{j}$$

(11) $$B_{j\prime }=\arg\max i{\;C}_{j}$$

The thickness was calculated by using the position vectors of two layers as:

(12) $$N=|B^{L1}-B^{L2}|$$

The thickness of the vein is a row vector of size 1 × *n*, *n* depending on the spatial extent of the region of interest of the vein.

To analyze the correlation between the predicted and the ground truth thickness values calculated using Equation (12), we consider the error values obtained on test data by calculating standard thickness error metrics: mean square error (MSE), mean absolute error (MAE), bias, standard deviation (SD), and root mean square error (RMSE) on the test dataset. The MAE and RMSE retain the same unit as the target thickness so directly give the measure of error; the MSE is related to the variance of the error; the bias is important in estimating systematic errors in prediction; the SD reflects the uncertainty associated with the predictions. They help in estimating not only the mean errors but also help in identifying systematic biases and assessing the impact of significant prediction errors. These metrics are crucial for comparing the accuracy and reliability of thickness measurement models. They were evaluated for UNET [19], SEGNET [27], RESUNET [23], PSPNET [26], DeepLab3+ [29], and TREE with respect to the ground truth (mask) values mentioned in Table 2. Apparently, TREE measures the thickness with fewest errors as depicted by all the metrics in Table 2. For SD, the SEGNET [27], and TREE perform nearly the same having the values 1.485 and 1.541, respectively. The errors calculated using the mean value of thickness per image give an estimate of how far the estimation lies from the real thickness value. Further, to calculate the thickness from the pixel values, we calculate the thickness (mm) T as:

(13) $$T=\frac{\mu }{n}\times N\times r$$

In Equation (13), *μ* = 9.61 μm (mean value) is the pixel resolution and *n* (=1.38) is the refractive index of tissue [43,44], N is the number of pixel values representing the thickness and *r* (=2) is the rescaling factor as the input image is resized to half before feeding to the prediction model. The thickness is calculated using Equation (13) for all the models along with TREE and with the ground truth thickness (Mask), tabulated in Table 3 as thickness (mm) averaged over the test data. The ground truth thickness (Mask) was calculated axially as it does not depend on any segmentation model outcomes and is directly calculated from expert-annotated masks which are highly accurate. It can be inferred that TREE estimates the thickness value very accurately. Further, in Table 3, we also present the number of parameters associated with each model. We can see that the fewest number of parameters are used in TREE as we have reduced from four encoder (and decoder) blocks to three as shown in Figure 1, compared to original UNET and other UNET-based architectures like RESUNET. The use of SPD does not burden the computational cost in terms of number of parameters; rather, it enhances the performance of the model, as can be inferred from results in Table 2 and Table 3.

To validate the robustness of the model, we cross-validate the performance of the proposed model on 200 B-scans acquired from the fifth volume acquired for another vein sample not used during training or validation. The segmentation performance of other models is compared to TREE on this cross-validation dataset in Table 4. It can be observed that the model outperforms the state-of-the-art methods, having the highest accuracy of 0.9242.

Furthermore, to demonstrate the effectiveness of transfer learning by incorporating the combination of encoder and dual decoders to predict the segmentation map and thickness value, we perform an ablation study. For this, we train several models on the venous volume dataset using the same procedure as described above in the sub-section: Experimental design and Setup. We train three different models: (i) En-De1 model where we first train the segmentation model followed by three dense and activation layers (as in De2) to extract the thickness of the vein; (ii) En-De1-De2 model includes encoder and both the decoders, trained simultaneously without any transfer learning, to predict the shape and thickness; (iii) En-De2 is trained to predict the thickness values exclusively without segmentation using the encoder and decoder; (De2) (iv) En-De2-De1 model takes the En-De2 from the previous (iii) model, where we fix the En weights and next we train only the decoder (De1); this model learns to regress the thickness value of the vein followed by segmentation; (v) NO-SPD—the model comprises En-De1-De2 trained similarly to TREE, but the SPD is replaced by bottleneck bridge layers as used in UNET [19]. Here, atrous and depthwise convolutions are replaced with regular convolutions with the same kernel size and stride, and keeping other layers and connections the same as in TREE. Here, in Table 5 we investigate the segmentation accuracy and the MSE for the thickness map on the test set. Here, the MSE is calculated for the thickness map which is a row vector containing the thickness of each column corresponding to every B-scan. It gives information about the thickness by calculating the mean error over all the columns (corresponding to a B-scan). This helps us to better understand the impact of transfer learning, dual decoders, and sequential training. The model (i) (En-De1) uses the same segmentation model as proposed in TREE; hence, they have a similar segmentation performance. The difference lies in the MSE for thickness estimation over the row vector as no transfer learning happens. In model (ii) En-De1-De2, the simultaneous training does not allow the model to predict the segmentation and thickness with great accuracy. The architecture of En-De2 is highly erroneous in the prediction of thickness and moreover it does not serve the purpose of this study to obtain a segmentation map and thickness from one network. Further, this model was trained sequentially (En-De2-De1) to predict the thickness followed by segmentation where it also performs poorly. The idea of initializing the training with the thickness prediction does not translate effectively to pixel-wise segmentation estimates. This model is unable to learn the global spatial information required for segmentation, hence making transfer learning ineffective. As the inherent idea behind UNET was to perform segmentation, it is therefore superior in capturing spatial hierarchies and context. These features are beneficial for segmentation and can be further fine-tuned to predict thickness values. Then, we also tested the model by replacing the SPD as described above, and it shows that the NO-SPD model also underperforms when compared with TREE. The best performance is given by TREE as seen in Table 5, significantly due to the transfer learning approach and the concept of SPD further enhances the performance. Hence, it is worth mentioning here that the idea of transfer learning in TREE, along with the innovation of the SPD module, results in attaining comprehensive information about the shape and thickness, using a single end-to-end network.

## 5. Discussion

The proposed model TREE was focused on a transfer learning approach using a combination of encoder and decoders for prediction of the segmentation map and the corresponding thickness value of the varicose veins. The images of the varicose vein were OCT images acquired using the SS-OCT system enabling high resolution along with depth information. The OCT B-scans of veins were taken as input images to train the model, which was optimized for two different predictions in two steps using the transfer of information between the two branches. This ensemble approach allowed the flow of information from one task to another. The accuracy can be attributed to the three components: (i) SPD module containing depthwise separable and atrous convolution for fine grained feature maps incorporating long-range spatial relationships; (ii) self-transfer learning which allows transfer of sensitive feature information; and (iii) residual connections for smoother convergence in a deep-layered architecture. The proposed model was compared to different state-of-the-art models by considering their performance in both the desired predictions (segmentation and thickness), for the varicose vein dataset. The proposed model establishes excellent performances for predictions.

To illustrate the influence of ensemble learning, we compared the thickness value obtained from the segmentation maps using the axial method to the ones of the proposed model. It was seen that even though all the scans in the volume dataset do not share the exact same features, the ability of the model to adapt to such dynamic changes makes it superior compared to others. The method is highly robust as it performs well even in the presence of specular reflections without pre-processing required to enhance the image quality. This results in highly accurate predictions, making it the most suitable approach that can be used for the mechanical characterization of veins. Hence, the precise and accurate outcomes of TREE opens room for further studies into physical properties of the veins. These mechanical properties can help analyze the causes of many diseases, and may aid the diagnosis and treatment procedures. In addition, the approach can also be extended to other applications where shape and thickness are of prime importance.

However, there are several limitations associated with the proposed work which can be improved. The OCT images are afflicted with speckle noise, which affects the sensitivity of the proposed model. Hence, denoising is crucial for enhanced learning and generalizability of the model. The OCT images suffer due to degradation in visibility of structures in depth, causing poor resolution for the lower boundary in veinous images. This problem can be addressed by using OCT systems with higher depth penetration and higher axial resolution for data acquisition. There is still room for improvement in terms of the optimization of the number of model parameters, and to predict more physical characteristics for veins. The multi-modal approach [45] can be also used to enhance the feature extraction process or to extract more biomarkers [46]; this can also allow us to overcome the limitations associated with OCT systems. In future. we plan to study the applicability of the proposed model in other segmentation and thickness prediction tasks, so that the model can be used for a broader range of applications.

## Figures and Tables

**Table 1:** List of metrics to evaluate the segmentation performance. The details include abbreviations, full-forms, formulas, and their significance. Here FP, FN, TP, TN denote False Positive, False Negative, True Positive, and True Negative, respectively.

Metric Group	Abbreviation	Full-Form	Equation	Significance
Spatial overlap	TNR	True Negative Rate	$\mathrm{TNR}=\frac{\mathrm{TN}}{\mathrm{TN}+\mathrm{FP}}$	Ratio of true background pixels in output to the overall background pixels in mask.
	TPR	Recall/True Positive Rate	$\mathrm{Recall}=\frac{\mathrm{TP}}{\mathrm{TP}+\mathrm{FN}}$	Ratio of true foreground pixels in output to the total foreground pixels in mask
	PPV	Precision	$\mathrm{Precision}=\frac{\mathrm{TP}}{\mathrm{TP}+\mathrm{FP}}$	Ratio of true foreground pixels predicted to total amount of foreground pixels in output
	F1	F1-score	$F1=2\;\times \;\frac{Precision\times Recall}{Precision+Recall}$	Overlap in the total number of pixels Mask and segmentation output
	IOU	Intersection Over Union/Jaccard Index	$\mathrm{IOU}=\frac{\mathrm{TP}}{\mathrm{TP}+\mathrm{FP}+\mathrm{FN}}$	Ratio of overlap and the union of pixels
Probabilistic	ACC	Accuracy	$\mathrm{ACC}=\frac{\mathrm{TP}+\mathrm{TN}}{\mathrm{TP}+\mathrm{TN}+\mathrm{FP}+\mathrm{FN}}$	Ratio of true pixels to total amount of pixels in output
	AUC	Area Under Receiver Operator	$\mathrm{AUC}=\frac{1+\mathrm{FPR}-\mathrm{TPR}}{2}$	Measure of the classification ability of the segmentation model

**Table 2:** Errors (pixels) in veins thickness estimation are represented using five metrics, namely MSE, MAE, Bias, SD, and RMSE on test set.

Method	MSE	MAE	BIAS	SD	RMSE
DeepLab3+	29.21	4.459	−2.155	4.957	5.405
UNET	14.36	3.115	0.794	3.706	3.790
PSPNET	56.900	6.4701	−4.010	6.388	7.543
RESUNET	34.224	4.7930	2.154	5.439	5.850
SEGNET	7.388	2.3684	−2.276	**1.485**	2.718
TREE	**2.409**	**1.109**	**0.184**	1.541	**1.552**

**Table 3:** Thickness (mm) for segmentation outputs of DeepLab3+ [29], UNET [19], PSPNET [26], RESUNET [23], SEGNET [27], ground truth (mask) and thickness predicted using TREE models. And number of parameters associated with each model (millions).

Method	DeepLab3+	UNET	PSPNET	RESUNET	SEGNET	MASK	TREE
Thickness (mm)	0.7486	0.7076	0.7744	0.6887	0.7502	**0.7168**	**0.7160**
Parameters (M)	44.5	31.04	48.70	43.5	29.45	**-**	**8.9**

**Table 4:** Cross-validation accuracy on DeepLab3+ [29], UNET [19], PSPNET [26], RESUNET [23], SEGNET [27], and TREE models.

Method	DeepLab3+	UNET	PSPNET	RESUNET	SEGNET	TREE
Accuracy	0.8325	0.8796	0.8471	0.8668	0.8295	**0.9242**

**Table 5:** Ablation study for segmentation and thickness.

Method	En-De1	En-De1-De2	En-De2	En-De2-De1	NO-SPD	TREE
Accuracy	0.9910	0.9282	-	0.6636	0.9514	0.9937
MSE (column)	0.1140	0.3554	58	58	0.2651	0.0537

## Data Availability

Dataset available on request from the authors.
